# Biodynamic lighting conditions preserve nocturnal melatonin production in pregnant women during hospitalization: A randomized prospective pilot study

**DOI:** 10.3389/fendo.2022.1043366

**Published:** 2022-12-08

**Authors:** Soyhan Bagci, Astrid Wieduwilt, Ebru Aileen Alsat, Jana Blickwedel, Brigitte Strizek, Christian Di Battista, Agnes Lachner, Herbert Plischke, Tamene Melaku, Andreas Müller

**Affiliations:** ^1^ Department of Neonatology and Pediatric Intensive Care, Children's Hospital, University of Bonn, Bonn, Germany; ^2^ Department of Obstetrics and Prenatal Medicine, University Hospital Bonn, Bonn, Germany; ^3^ Engineering and Architecture, University of Lucerne, Lucerne, Switzerland; ^4^ Applied Sciences, Munich University, Munich, Germany

**Keywords:** pregnant women, circadian rhythm, melatonin, human-centric lighting, biodynamic lighting

## Abstract

**Background and purpose:**

Maternal circadian rhythms are important for maintaining maternal and fetal homeostasis. The maternal circadian system coordinates the internal clock of the fetus with environmental lighting conditions *via* the melatonin signal. The intensity and wavelength of daylight influence nocturnal melatonin production. This study aims to evaluate the effect of environmental lighting conditions on melatonin production in pregnant women with reduced mobility during hospitalization.

**Methods:**

We installed a human-centric lighting system with biodynamic effects (BDL, biodynamic lighting) in the patient rooms. The pregnant women in the patient rooms with standard indoor conditions served as a control group. The illuminance (lux) and dose of effective circadian irradiation (Hec) were recorded every 10 seconds by light dosimeters (Lucerne University, Switzerland) attached to the patients` clothing.

**Results:**

We analyzed the illuminance status of 47 pregnant women with a median (IQR) gestational age of 29.9 (25.4-32.3) weeks of gestation. The median illuminance in the control group was significantly lower (p<0.05) than in the BDL group in the morning and afternoon from day 1 to 5. BDL patients had a significantly higher effective circadian irradiation in the morning. The effective circadian irradiation showed a significant daily rhythm only in the BDL group. The BDL group had a significantly higher melatonin production on day 3 (p=0.006) and day 5 (p=0.012) than the control group median (IQR) nocturnal 6-Sulfatoxymelatonin excretion 15840 (10140-22160) ng/12h vs. 6141 (2080-11328) ng/12h on day 3 and 18780 (11320-23562) ng/12h vs. 6380 (3500-17600) ng/12h on day 5).

**Conclusion:**

We have demonstrated that dramatically altered lighting conditions of hospitalized pregnant women may be optimized by installing biodynamic lighting systems in the patient rooms resulting in the maintenance of nocturnal melatonin production in pregnant women.

## Introduction

Circadian rhythms regulated by a circadian timing system are essential for many physiological and psychological functions in humans. Pregnancy is a physiological process that requires the synchronized adaptation of multiple organ systems. The circadian timing system plays a vital role in regulating several physiological processes during pregnancy, including blood pressure, blood flow, body temperature, glucose availability, rhythms of uterine contraction, hormone levels, and intra-amniotic fluid pressure (1, 2). These rhythmic activities are essential for maintaining maternal and fetal homeostasis. Although the genetic background and environmental stimuli, such as temperature, sound, food, and social cues, seem to influence circadian phase control, the daily environmental light-dark cycle is the primary cue for circadian entrainment in humans (3). Light effects on circadian rhythms depend on the length of the day, duration of exposure to daylight, and timing of exposure to artificial light (4).

Natural daylight is an essential factor regulating the activity of the internal biological clock. The pineal gland is a central structure in the circadian timing system and the primary source of the hormone melatonin. As a small lipid and water-soluble indoleamine molecule, melatonin can easily cross membrane barriers. Thus, melatonin regulates many physiological processes, including circadian rhythms, immune functions, antioxidative processes, cardiovascular functions, endocrine regulation, sleep physiology, mood regulation, seasonal reproductive physiology, and body temperature homeostasis (5). The synthesis and secretion of melatonin are highly rhythmic, exhibiting maximal levels in the middle of the night and a gradual decline towards dawn. This circadian fluctuation of melatonin is crucial for the synchronization of the biological day rhythm (5).

There is general agreement that the maternal circadian system coordinates the internal clock of the fetus with environmental lighting conditions *via* the melatonin signal (1, 6). Previous studies have demonstrated that the maternal circadian rhythms during perinatal development are essential for optimal fetal growth, perinatal neurodevelopment, and the preparation of the circadian timing system for later independent life and normal metabolic functions in adulthood (7, 8). There is also clear evidence supporting the role of melatonin in a successful pregnancy (9). Therefore, any prolonged alteration may have detrimental effects on fetal development and maternal health (2, 10).

Approximately 20% of pregnant women are recommended bed rest sometime during pregnancy to improve maternal/fetal health outcomes for high-risk pregnancies (11, 12). These high-risk pregnant women especially hospitalized pregnant women, spend most of their time indoors, resulting in a decrease in exposed daytime outdoor light and an increase in artificial light at night (ALAN) (13). ALAN imposes negative impacts on many organisms, including increased alertness, depression, elevated risk of hormone-dependent cancers, circadian phase disruption and sleep disorders in humans (14). It is known that not only ALAN but also less light during the daytime negatively impact circadian physiology. Recently, we demonstrated the dramatically altered environmental lighting conditions in women with high-risk pregnancies during their stay in hospital (15). In this randomized prospective pilot study, we aimed to evaluate the effect of environmental lighting conditions on melatonin production in hospitalized pregnant women with reduced mobility during hospitalization. We hypothesized that altered lighting conditions, such as inadequate daylight exposure or constant environmental lighting, may affect nocturnal melatonin production in pregnant women during their stay in hospital. We tested our hypothesis using a human-centric lighting (HCL) system with biodynamic effects in the patient rooms.

## Materials and methods

This prospective study involved 70 pregnant women hospitalized between January 2017 and February 2018 in the maternity ward at University Hospital Bonn, Germany. In accordance with the Declaration of Helsinki, the study was approved by the Ethics Committee of the University of Bonn (373/16). Written informed patient consent was obtained. Inclusion criteria were having healthy fetus, informed consent to participate in the study, no drug addiction, and having no significant psychological, sleeping, or metabolic disorder, that may have impact on melatonin secretion. Women hospitalized due to abortion or feticide were excluded from the study.

### Lighting conditions in the patient rooms

We installed human-centric lighting (HCL) system with biodynamic effects (abbreviated as BDL, biodynamic lighting) in 2 patient rooms. Since the reconstruction of the conventional lighting in the patient rooms was not possible or practical, we converted a wall-mounted luminaire ZERA BED (Derungs Licht AG, Gossau, Switzerland) to free-standing luminaires (**Supplementary Figures 1** and **2**). This also offered the flexibility of moving the BDL system to another room together with the patient if necessary. Patient beds in these rooms were lit between 7:30 and 21:30 by a standard biodynamic lighting scenario of ZERA BED. Time interval was determined according to routine working conditions (visit etc.) and daily life rhythms of pregnant women in the maternity ward. The patient rooms with standard lighting conditions were used to control patients. Control patients were informed that they could use a conventional lighting system for rooms as needed. Exposed illuminance (lux) and effective circadian irradiation dose (16, 17) (Hec, J/m^2^) were measured and recorded every 10 seconds by light dosimeters (LDM, produced for research purposes by the University of Lucerne, Switzerland) attached to the patients’ clothing. The patients filled in a questionnaire every day, a study diary, to get more details about their daily routines regarding strolls outdoor, the time of putting on the LDM in the morning and turning it off in the evening.

### Analysis of nocturnal melatonin production

To evaluate the nocturnal melatonin production in pregnant women *via* a noninvasive and non-stressful tool, we collected urine samples from 8 pm to 8 am on day 1 (to evaluate basis nocturnal melatonin status), day 3 (to evaluate whether nocturnal melatonin production is changed in 48h after hospitalization), and day 5 (to evaluate whether nocturnal melatonin production may adapt the suboptimal environmental condition). Excreted urine volume in 12 h was measured to calculate the cumulative nocturnal 6-Sulfatoxymelatonin (aMT6s) excretion. The samples were centrifuged at 3000 g for 5 min within one hour and stored at -80°C until analysis.

The urinary concentrations of aMT6s were determined using a direct enzyme-linked immunosorbent assay (IBL-International, Hamburg, Germany) in the Laboratory of the Department of Neonatology, University of Bonn, Germany (18). The assay procedure followed the basic principle of a competitive ELISA. The results were expressed as ng/mL. The mean recovery of aMT6s was 105.8%, and the assay’s sensitivity was 1.0 ng/mL. The intra-assay variation was 5.2% at 5.8 ng/mL and 12.2% at 204 ng/mL. The inter-assay variation was 5.1% at 12.4 ng/mL and 14.9% at 220 ng/mL. The mean recovery of aMT6s was 105.8%, and the assay’s sensitivity was 1.0 ng/mL. All samples were analyzed in duplicate. The night aMT6s excretions were calculated by multiplying the measured aMT6s concentrations by the 12-h urine volumes and expressed as ng/12h.

### Statistical analyses

The patients were randomly assigned to the study. In the clinic, patients are usually admitted to a room where there is a free bed. To evaluate the effect of altered lighting conditions, we chose to divide the women into two groups: the BDL group that included the women who were hospitalized in a patient room with the BDL system and another group of women who were hospitalized in a patient room with standard lighting conditions but spent more than 60 minutes (measured illuminance more than 1000 lux) outdoors per day. The control group included pregnant women in the patient rooms with standard lighting and less than 60 minutes of outdoor mobility. Data from the pregnant women who were hospitalized for more than 3 days were included in the statistical analysis. To compare the median illuminance and effective circadian irradiation between both groups, we divide the recorded daytime data in 5 intervals: the morning (0700-1200), the afternoon (1200-1700), early evening (1700-2130), late evening (2130:0000), and night (0000-0700). We have considered the different conditions such as the start of the work of nursery, sunrise in Bonn (in 4 seasons) etc. The statistical analyses were performed using SPSS 25.0 (SPSS Inc., Chicago, IL, USA). The variables were tested for normality using the Kolmogorov-Smirnov test. Because the values were not normally distributed, we used the two-tailed non-parametric Mann–Whitney-U-test for the group comparison. The non-parametric Friedman and Wilcoxon signed-rank tests for paired samples were used for the inferential statistics. Spearman’s rank correlation coefficients test was used to analyze associations between parameters. All data are presented as the median (interquartile range (IQR)). For all analyses, p values of less than 0.05 were considered statistically significant.

## Results

Forty-seven of the 70 pregnant women remained at least three days in the hospital. Twenty-three women were excluded from the analysis: 17 women were discharged in the first 48 hours from the hospital, two women collected no urine samples according to the study protocol, and LDM in 4 women was not correctly initialized. The illuminance status of 47 pregnant women was analyzed (**Table 1**). The BDL group included 27 patients (25 patients hospitalized in the patient room with a BDL system and 2 patients hospitalized in a room with standard lighting but spent more than 60 minutes (measured illuminance more than 1000 lux) outdoors per day. The control group included 20 patients. There was no statistical difference between the BDL and the control group for GA (median GA; 30.6 (25.8-33.0) vs. 28.6 (24.7-32.0), p=0.220).

**Table 1:** Study characteristics.

Current maternal characteristics	
Maternal age (years), median (IQR)	30.1 (25.4-33.3)
Gestational age (weeks), median (IQR)	29.9 (25.4 -32.3]
Cervical insufficiency/Short cervical length, n (%)	22 (47)
Premature labor, n (%)	17 (36)
Vaginal bleeding, n (%)	13 (28)
Twin pregnancies, n (%)	9 (19)
Fetal anomalies, n (%)	6 (13)
IUGR/placenta insufficiency, n (%)	4 (9)
Gestational diabetes, n (%)	3 (6)
Hypertension, n (%)	3 (6)

IUGR, intrauterine growth restriction.

The median illuminance in the control group was significantly lower than in the BDL group in the morning (0700-1200) and afternoon (1200-1700) from day 1 to 5 (**Table 2**). The BDL group had a significantly higher effective circadian irradiation in the morning. The effective circadian irradiation showed a significant daily rhythm only in the BDL group between 0700 and 2130 (**Table 3**). The median illuminance and effective circadian irradiation in the late evening and at the night were not statistically significant different between groups (p>0.05) (not presented in the Tables). We also evaluated the duration of exposed illuminance above 300 lux. Compared to the BDL group, the median duration of exposed illuminance above 300 lux was significantly lower in the control group both in the morning and the afternoon (0 min (0-36) vs. 112 min (38-190) and 20 min (0-65) vs. 128 min (48-215), p <0.001).

**Table 2:** The exposed illuminance (Lux) of the patients in the group with a biodynamic lighting system (BDL) and the control group during the inpatient stay.

		Illuminance, lux		
Day	Time	BDL (n=27)	Control (n=20)	p Value
**Day 1**	0700-1200	279 (130-361)	33 (21-56)	<0.001
	1200-1700	295 (204-499)	59 (20-85)	<0.001
	1700-2130	121 (62-214)	28 (9-76)	0.002
**Day 2**	0700-1200	249 (76-329)	43 (17-109)	0.002
	1200-1700	275 (172-437)	68 (50-118)	<0.001
	1700-2130	91 (37-196)	30 (7-89)	0.031
**Day 3**	0700-1200	233 (81-389)	46 (23-145)	0.002
	1200-1700	308 (213-386)	95 (26-205)	<0.001
	1700-2130	80 (46-135)	47 (9-133)	0.280
**Day 4**	0700-1200	241 (128-327)	69 (34-143)	0.001
	1200-1700	201 (107-348)	98 (43-152)	0.017
	1700-2130	120 (68-301)	22 (3-200)	0.047
**Day 5**	0700-1200	308 (133-431)	75 (60-195)	0.011
	1200-1700	262 (175-354)	95 (55-133)	<0.001
	1700-2130	123 (51-199)	53 (13-89)	0.162

The data are expressed as the median (IQR).

**Table 3:** The dose of the effective circadian irradiation (Hec) of the patients in the group with a biodynamic lighting system (BDL) and the control group during the inpatient stay.

		Effective circadian irradiation dose (Hec, J/m^2^)		
Day	Time	BDL(n=27)	Control(n=20)	p Value
**Day 1**	0700-1200	760 (386-1399)	124 (34-314)	<0.001
	1200-1700	180 (110-398)	84 (13-534)	0.098
	1700-2130	93 (51-177)	52 (7-200)	0.292
p Value*		0.002; <0.001; 0.012	ns	
**Day 2**	0700-1200	628 (336-1415)	153 (67-509)	0.001
	1200-1700	262 (122-424)	237 (36-438)	0.464
	1700-2130	117 (43-214)	39 (11-189)	0.111
p Value*		<0.001; <0.001; <0.001	ns	
**Day 3**	0700-1200	875 (626-1230)	258 (35-692)	<0.001
	1200-1700	371 (196-1202)	90 (22-229)	0.001
	1700-2130	93 (60-120)	60 (4-130)	0.175
p Value*		0.023; <0.001; <0.001	ns; 0.009; 0.025	
**Day 4**	0700-1200	746 (472-1080)	235 (79-440)	<0.001
	1200-1700	268 (113-450)	100 (26-482)	0.158
	1700-2130	120 (68-301)	91 (1-230)	0.165
p Value*		0.001; <0.001; ns	ns; 0.030; ns	
**Day 5**	0700-1200	955 (775-1183)	204 (120-499)	0.001
	1200-1700	219 (138-438)	69 (31-540)	0.101
	1700-2130	117 (93-148)	43 (26-179)	0.141
p Value*		0.001; <0.001; 0.002	ns; 0.028; ns	

The data are expressed as the median (IQR).

* p Value: 0700-1200 vs. 1200-1700; 0700-1200 vs. 1700-2130; 1200-1700 vs. 1700-2130, ns, non-significant.

Although nocturnal melatonin production on day one did not differ significantly between the two groups, the patients in the BDL group had a significantly higher melatonin production on day 3 and day 5 than the control group (**Table 4**). Spearman correlation analysis showed no significant effect of the age of the pregnant women or their gestational age on the nocturnal melatonin production (p>0.05).

**Table 4:** The nocturnal 6-Sulfatoxymelatonin (aMT6s) excretion in the urine of patients in the group with a biodynamic lighting system (BDL) and the control group during inpatient stays.

	aMT6s Excretion in Urine (ng/12h)		
**Day**	BDL (n=27)	Control (n=20)	p Value
**First day**	12600 (6222-19775)	10490 (5080-19561)	0.715
**Day 3**	15840 (10140-22160)	6141 (2080-11328)	0.006
**Day 5**	18780 (11320-23562)	6380 (3500-17600)	0.012

The data are expressed as the median (IQR).

## Discussion

Our study presents the first information about the effect of dramatically altered environmental lighting conditions and the use of biodynamic lighting systems on nocturnal melatonin production in high-risk pregnant women during their stay in hospital. At least 3 days of bed rest in the hospital dramatically reduce illuminance both in the morning and afternoon for pregnant women resulting in a decreased melatonin production at night. The installation of HCL systems with BDL-effects provides higher illuminance and effective circadian irradiation with a significant daily rhythm, which helps maintain nocturnal melatonin production in pregnant women.

Preclinical and clinical studies suggest that the human fetus shows 24-hour rhythms in several physiological functions, including fetal breathing movements, fetal heart rate, fetal limb movements, and the synthesis of hormones such as cortisol (19–22). There is general agreement that the fetal biological clock receives information about environmental lighting conditions either *via* the maternal melatonin or by other signals from the maternal circadian system. Because of its highly lipophilic nature, maternal melatonin can rapidly crosse from maternal blood to the fetal circulation, permitting maternal photoperiodic information to generate day/night differences in the fetus (23, 24). Cyclic melatonin secretion is needed a day/night light cycles in an acceptable manner and duration. Therefore, altered lighting conditions, such as insufficient daylight, constant environmental lighting, reduced environmental differences between day and night, and ALAN may result in disrupted nocturnal melatonin production (25). We observed several pathological daytime lighting conditions in the control group. The median exposed illuminance was not only insufficient but also well below the recommended EU standard of 100 lux. The duration of exposed illuminance above 300 lux was significantly lower in the control group both in the morning and afternoon. Another problem was both the low and unchanged effective circadian irradiation in the control group. These pathological daytime lighting conditions resulted in decreased melatonin production in the control group. We observed several factors related to low illuminance and the spectrum of exposed light in our control patients. The main problem in our patient rooms, like most other clinics, was architectural and the existing lighting systems, which had not been designed for the specific needs of hospital patients. To prevent direct sunlight exposure, the women with their bed next to the window tended to close the curtains on sunny days, causing insufficient light exposure. Because of the uncomfortable illuminance of recessed luminaires for the patients, often forced to the horizontal position in the bed, the women tend to use indirect lighting from the headboard with very low illuminance. However, our analysis showed that these lighting conditions provided an advantage by reducing exposure to ALAN. The pregnant women were not exposed to ALAN between 2130 and 0700 (median (IQR) exposed illuminance was 0 (0-0) lux in both groups). Because of the settings of our BDL systems, patient rooms were lit only between 7:30 and 21:30h. Therefore, the effect of ALAN was minimal on nocturnal melatonin production in our collective.

Outdoor light intensities can be as great as 100,000 lux in direct sunlight and usually ~25,000 lux in full daylight during the daytime. Theoretically expected indoor light intensities in closed rooms are only 200 to 300 lux without and about 500 lux with artificial lighting (26). In recent years, there has been a certain trend in the issue of the impact of indoor lighting conditions on the emotional wellbeing, comfort, health, and productivity of people. In a new concept, defined as “Human Centric Lighting” (HCL), the keyword is “human”, that is a person, and their health is the focus of attention. However, HCL systems should offer biodynamic solutions based on the person’s circadian rhythm, determined by the sun’s spectrum, to help maintain the alignment of circadian biological rhythms and basic processes even under artificial lighting. Our recent study has emphasized dramatically altered environmental lighting conditions on women with high-risk pregnancies during their stay in hospital. Their exposure to light is significantly lower than natural daylight levels and below the recommended EU standard of 100 lux. An illuminance exposure above 300 lux was reached only during 11.5% of the inpatient time in the morning and 18.1% in the afternoon 15. In this study, we were able to increase the doses and the duration of exposed adequate illuminance with the installation of BDL systems in the patient room. The pregnant women in the BDL group were exposed to more than 100 min above 300 lux illuminance both in the morning and afternoon. In contrast, the pregnant woman in the control group was rarely exposed to more than 300 lux, demonstrating a total daylight deprivation. Another effect of BDL systems was to provide a significant daily rhythm for effective circadian irradiation. Highly specialized light-sensitive retinal photoreceptors are the primary means the central circadian clock is synchronized to environmental time. Both low daylight illuminance and the absence of oscillation in daily effective circadian irradiation increase retinal sensitivity to blue light and decrease circadian rhythm stability (27).

In recent years several researchers have pointed to the importance of a normal circadian rhythm and melatonin levels for optimal reproductive physiology and fetal programming during pregnancy (for more information, see reviews (1, 28, 29). Epidemiological studies have revealed an association between reduced melatonin levels and consequent chronodisruption during pregnancy and an increased risk of adverse pregnancy outcomes such as miscarriage, preeclampsia, chronic placental insufficiency, preterm delivery, and low birth weight (30–32). Moreover, disrupted maternal circadian rhythms and reduced melatonin levels result in inappropriate fetal programming, increasing the risk of cardiovascular, metabolic, neuroendocrine, and neuropsychiatric disorders in adulthood (1, 28, 29). Based on current scientific data, we tried in this pilot study to optimize environmental lighting conditions by using BDL systems to avoid circadian disruption resulting in reduced nocturnal melatonin production. Although we could not achieve a continuous median illuminance of more than 300 lux, our results demonstrated that illuminance of more than 200 lux, both in the morning and afternoon with a daily rhythm of effective circadian irridation, can maintain nocturnal melatonin production in pregnant women.

Our study has some limitations. We had no information about the last-day illuminance status of patients included in our study. It is known that high-risk pregnant women tend to stay at home. Therefore, it is possible that some pregnant women from both groups were exposed to low illuminance if they had stayed at home with bad lighting conditions before their hospitalization. Because we could not measure lighting conditions in their home, we had to ignore their last-day histories and use as a basis their melatonin production on the first night in the hospital to compare in the course. The nocturnal melatonin production in the BDL group tended to increase, while in the control group, it decreased. This may be explained by the better lighting conditions in the BDL rooms and the poor lighting conditions in the control rooms compared to the home lighting conditions of some patients. Another technical problem was achieving more than 300 lux in the patient rooms with BDL systems. The ZERA BED luminaires used in the study had an indirect lighting component that radiated the light from the wall and reflected it into the room. This was necessary for patient comfort to avoid continuous light exposure to the eyes of the pregnant women in bed. Moreover, we could not design these BDL systems to suit the architectural plans and lighting requirements of every patient room. Therefore, the median exposed illuminance to our patients was between 200 and 310 lux. Although these illuminance values could maintain nocturnal melatonin production in the normal daily range of pregnant women, an illuminance of around 500 lux may be more effective for hospitalized pregnant women. Therefore, future studies with different illuminance and biodynamic lighting scenarios are needed to establish ideal environmental conditions, not only for pregnant women but also for all long-hospitalized patients, especially in intensive care units.

## Conclusions

Our findings demonstrated that altered lighting conditions for hospitalized pregnant women might be optimized with the installation of biodynamic lighting systems in the patient rooms resulting in the maintenance of their nocturnal melatonin production. Our results support the importance of maintaining the natural environmental lighting conditions to balance maternal and fetal melatonin homeostasis during pregnancy. Therefore, it is crucial to plan new clinics with a concept of HCL with biodynamic effects to reduce the risk and impact of chronodisruption in pregnant women and their fetuses during pregnancy.

## Data availability statement

The original contributions presented in the study are included in the article/**Supplementary Material**. Further inquiries can be directed to the corresponding author.

## Ethics statement

The studies involving human participants were reviewed and approved by Ethics Committee of the University of Bonn (No: 373/16). The patients/participants provided their written informed consent to participate in this study.

## Author contributions

All authors have read and complied with the authorship criteria. They have made a significant contribution to the manuscript and accept the responsibility for the study protocol and the presented results. SB, HP, and AM designed research; SB, AW, EA, BS, and TM conducted research; SB, AL, CB, and TM analyzed data; AM critically reviewed and revised the manuscript; SB and EA wrote the paper and approved the final manuscript as submitted. All authors read and approved the final manuscript.

## Supplementary material

Supplementary Figure 1 Schematic 3D representation of the patient room with biodynamic lighting (Zera Bed, Derungs Licht AG, Gossau, Switzerland). The biodynamic lamp was attached from the ground at a height of 2 m. The distance from the lamp to the eye of the lying subject is 1.4 m. The indirect light share takes up an area of 9 m^2^.

Supplementary Figure 2 Indirect lighting of a patient bed *via* human-centric lighting (HCL) system with biodynamic effects (Zera Bed, Derungs Licht AG, Gossau, Switzerland).
