# Supplementary figures and images for: Biodynamic lighting conditions preserve nocturnal melatonin production in pregnant women during hospitalization: A randomized prospective pilot study
Source: Front Endocrinol (Lausanne). 2022 Dec 8;13:1043366. doi: 10.3389/fendo.2022.1043366 (PMC9774480; doi:10.3389/fendo.2022.1043366)

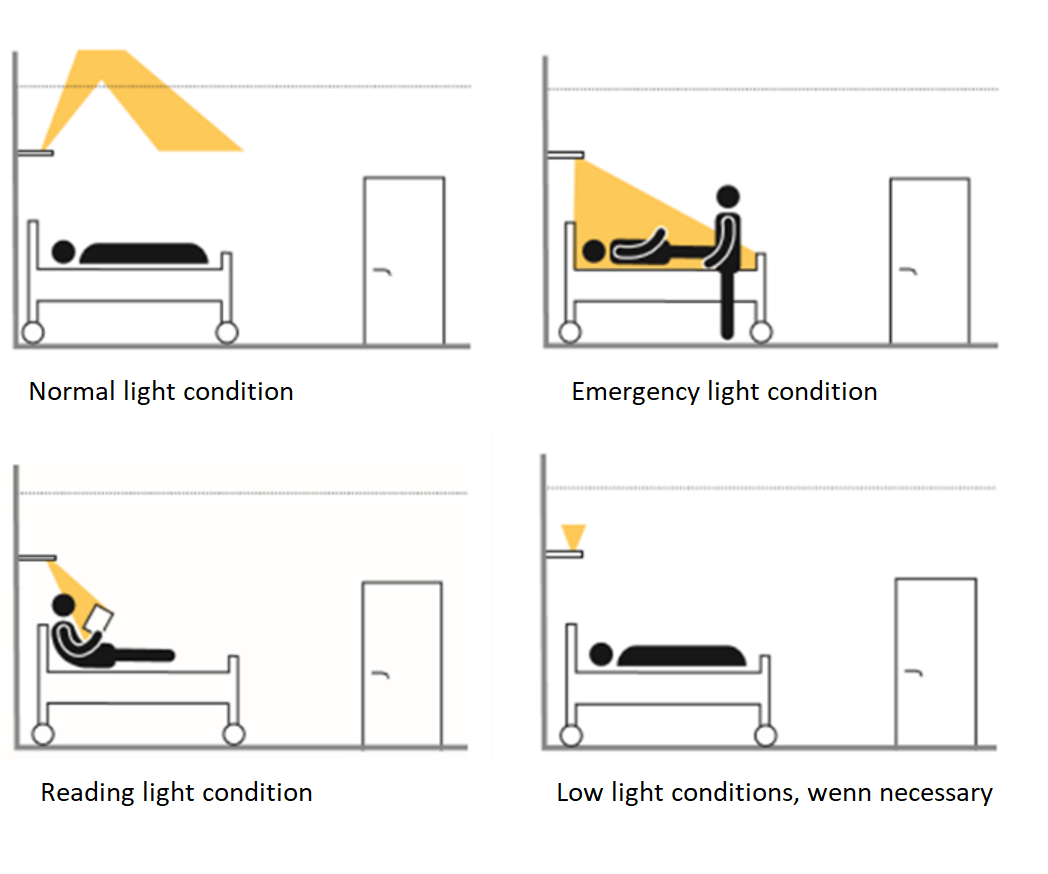

Supplement: Supplementary Figure 1 — Schematic 3D representation of the patient room with biodynamic lighting (Zera Bed, Derungs Licht AG, Gossau, Switzerland). The biodynamic lamp was attached from the ground at a height of 2 m. The distance from the lamp to the eye of the lying subject is 1.4 m. The indirect light share takes up an area of 9 m2. [file Image_1.tif]

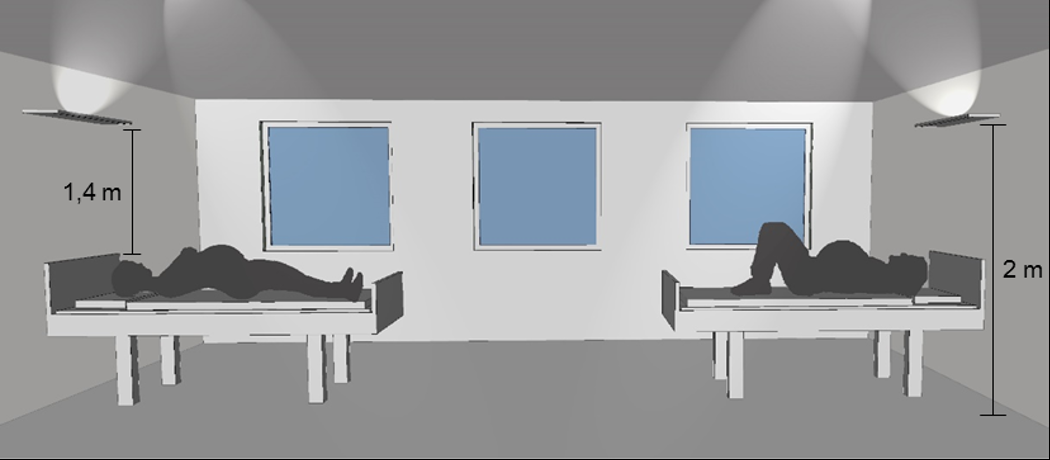

Supplement: Supplementary Figure 2 — Indirect lighting of a patient bed via human-centric lighting (HCL) system with biodynamic effects (Zera Bed, Derungs Licht AG, Gossau, Switzerland). [file Image_2.tif]
